# Do Emotional Laborers Help the Needy More or Less? The Mediating Role of Sympathy in the Effect of Emotional Dissonance on Prosocial Behavior

**DOI:** 10.3389/fpsyg.2019.00118

**Published:** 2019-02-07

**Authors:** Yun-na Park, Hyowon Hyun, JiHoon Jhang

**Affiliations:** ^1^School of Business, Hanyang University, Seoul, South Korea; ^2^Spears School of Business, Oklahoma State University, Oklahoma, United States

**Keywords:** emotional labor, emotional dissonance, surface acting, deep acting, sympathy for others’ feeling, emotional exhaustion, prosocial behavior

## Abstract

Despite the growing body of research on emotional labor, little has been known about the social consequences of emotional labor. Drawing on emotional dissonance theory, the authors investigate the relationship between the felt emotional dissonance and prosocial behavior (e.g., donation to a charity). Findings from multiple studies suggest that higher emotional dissonance serially influences perceived lack of control, emotional exhaustion, lowered sympathy for others’ feeling, and subsequently lower willingness to help others. When individuals are asked to recall their past experiences of emotional dissonance, they expressed lack of control and emotional exhaustion (Study 3), lower sympathy for others’ feeling (Studies 1, 3), and subsequently become less willing to help others both in their intention (Studies 2A and 3) and with actual money (Study 2B). Further, this negative relationship is moderated by the display rules (i.e., surface acting vs. deep acting, Study 3). Managerial and public policy implications are discussed.

## Introduction

According to the Bureau of Labor Statistics (2016), about 80% of total U.S. workers are employed by the service sector, and employment in the service sector is projected to increase over 12% between 2008 and 2018 (Bureau of Labor Statistics, 2016). Situations in other developing countries do not differ much (The World Bank, 2016). As the proportion of service-sector employees increases, it becomes more important to understand and manage these service-sector employees.

One characteristic peculiar to service-sector employees is their frequent contacts with customers. Therefore, previous research has emphasized the importance of managing these employees because evaluation of a service firm often depends on how customers perceive the interaction they have with the frontline employees (Bitner, 1990; Abraham, 1998). For instance, Babakus et al. (2009) showed that frontline employees play a crucial role not just in the service delivery but also in customer relationship building. More important, research shows that the expressed emotions of service providers heavily influence customers’ perceptions of service quality (Abraham, 1998). For this reason, many organizations in the service industry attempt to control the way their employees display their emotions. Consequently, the service-sector employees are very often required to express certain emotions different from their true ones. The term ‘emotional labor,’ first coined by Hochschild (1979, 1983) was originated from describing a situation wherein employees are required to display emotions that may differ from what they truly feel at the moment especially in the context of client contacts.

Despite its positive impact on customers’ perceptions of service quality, the enforced emotional display rules are known to have negative impacts on both individual and organizational well-being (Grandey, 2000). To name a few, a body of research in emotional labor consistently found that emotional laborers tend to have lower identification with the organization, lower job involvement, lower job satisfaction, higher work stress, and lower well-being (Pugliesi, 1999; Schaubroeck and Jones, 2000; Cho et al., 2013). Because of its significance, much research efforts have been devoted to examine the antecedents, dimensions, and consequences of emotional labor (e.g., Rafaeli and Sutton, 1987; Morris and Feldman, 1996; Groth et al., 2009).

Common in the aforementioned works is that most research has focused only on the outcome variables related to organizations’ performance. That is, the majority of works has been done from the perspectives of organizations. In spite of its practical and theoretical significance of this topic, there is a dearth of research on this topic from consumers’ perspectives. To our best knowledge, no research efforts have been devoted to examine the behaviors of emotional laborers as *consumers*. Although this may not be directly related to organizational well-being such as job performance or job retention rate, we argue that it would have even bigger implications to the society given that the majority of employees are to some extent considered as emotional laborers (Bureau of Labor Statistics, 2016). Therefore, it is very timely and important to investigate how these emotional laborers behave as consumers. For instance, how these people would treat other service workers (e.g., emotional laborers) when they become customers? Would they be sympathetic for others’ feeling more or less? why? And what would be the consequences of that? These are the questions of interest the present research would address.

The purpose of this research is to understand the psychology of emotional labor and its consequences beyond the organizational boundary. Specifically, we examine how emotional laborers would differ in their degree to feel sympathy toward others (or others’ feeling) and how this difference results in their pro-social intention and actual pro-social behavior. By examining these, we would like to shed light on the societal consequences of emotional labor, which has been neglected in the previous literature.

## Theoretical Background

### Emotional Labor as Regulatory Behavior

Since Hochschild (1983) first coined the term ‘emotional labor’ as “*the management of feeling* to create a publicly observable facial and bodily display” (p. 7), many researchers have attempted to grasp the concept of emotional labor by providing their own definition for the term. For instance, Ashforth and Humphrey (1993), focusing more on the observable behavior rather than the management of feeling, defined emotional labor as “*the act* of displaying appropriate emotions with the goal to engage in a form of impression management for the organization” while Morris and Feldman (1996) defined it as “the effort, planning, and control needed to express organizationally desired emotion during interpersonal transactions.” Grandey (2000) defined emotional labor as *the process of faking and suppressing their true emotions* to follow the guidelines imposed by organizations.

Although the exact wording varies by scholars, what is common among various definitions is the notion of *emotion regulation* for the organization; emotional laborers must regulate their emotions to achieve bigger goals (i.e., organizational goals). Therefore, Diestel and Schmidt (2011) think emotional labor as a goal-directed regulatory behavior.

### Deep Acting and Surface Acting

One important (and implicit) assumption implied by the term ‘emotion regulation’ is that some emotions have to be regulated because, without being regulated, they might not be appropriate from the perspectives of organizations. For instance, if there is a conflict between the emotions an employee wants to express and the emotions an organization wants their employees to express, the employee must regulate emotions.

According to Gross (1998), people can regulate emotions in two ways; focusing on either the precursors of emotions (e.g., the situation) or the observable signs of emotions (e.g., facial expressions). In the first emotion regulation technique, employees adjust their emotional responses to the situation by *modifying the way they perceive the situation*. By either thinking about events that call up specific emotions needed in the situation or reappraising the objects or situations, employees try to modify their thoughts and feelings with the goal to make the expression more genuine. This type of emotion regulation process corresponds to the emotional labor concept of ‘deep acting’ (Hochschild, 1983).

By contrast, in the second emotion regulation technique, employees just manipulate emotional expressions of their responses to the situation without adjusting the perception of the situation. By either faking the expressions entirely or adjusting the intensity of the displayed emotions, employees try to *modify their expressions without changing their thoughts or feelings*. This type of emotion regulation process corresponds to the emotional labor concept of ‘surface acting’ (Hochschild, 1983).

When an employee is engaged in this second type of emotion regulation technique (i.e., surface acting), the employee recognizes that the emotion he/she expressed differs from the emotion he/she actually felt, and consequently feels emotional discrepancy. This perceived discrepancy is called ‘emotional dissonance’ (Hochschild, 1983; Abraham, 1998; Kruml and Geddes, 2000). In the literature on emotional labor, it is a widely held notion that surface acting is linked with emotional dissonance (e.g., Ashforth and Humphrey, 1993; Deng et al., 2017).

### Emotional Dissonance and Perceived Control

Emotional dissonance, in many aspects, is analogous to cognitive dissonance (Festinger, 1957). Specifically, the following three propositions are noteworthy. According to the cognitive dissonance theory (Festinger, 1957), (1) people are sensitive to the discrepancy between their belief and action, (2) people, when recognizing the discrepancy, are motivated to resolve the discrepancy, and (3) people, in attempting to resolve the discrepancy, usually trace back to why the discrepancy arose (e.g., Ben, 1967). When we apply these to the context of emotional labor, an employee engaged in surface acting can recognize the discrepancy between their felt and expressed emotions and be motivated to resolve the discrepancy. In attempting to resolve the discrepancy, the employee would trace back to why the perceived emotional discrepancy arose. The reason is obvious; they had to follow the display rule enforced by the organization. Therefore, we argue that this reasoning leads the employee to perceive that she has no control over the way she expresses her true feeling, which may result in a generalized lower sense of control^1^ (or perceived lack of autonomy).

Organizational behavior literature on the perceived control has equivocally reported positive relationship between high levels of perceived control and job-related variables (e.g., Spector, 1986; O’driscoll and Beehr, 2000; Thompson and Prottas, 2005). For instance, Spector (1986) showed that employees’ high levels of perceived control positively affect not only organizational (e.g., job satisfaction, commitment, involvement, performance, and motivation) but also individual facets (e.g., emotional distress, role stress, absenteeism, and turnover rate). In a similar vein, Thompson and Prottas (2005) found that job autonomy and perceived control are positively associated with numerous organizational outcomes. These findings imply that lower (vs. high) perceived control that results from emotional dissonance, would have negative impacts on job-related variables.

More relevant to our theorizing are the findings by Muraven et al. (2007) that demonstrate the relationship between the lowered sense of control and resource depletion. In their studies, Muraven et al. (2007) randomly assigned employees to either controlling situation (i.e., working environment where everything was structured so that no autonomy is allowed) or autonomy supportive situation (i.e., working environment where maximum autonomy is allowed) and examined employees’ degrees of depletion. The results show that people tend to be depleted more when they were working in the controlling situation. This suggests that when people perceive lack of control over the situation they are working in, they are more likely to be depleted. Taken together, we argue that emotional dissonance leads to resource depletion via lowered perceived control.

### Emotional Exhaustion and Sympathy for Others’ Feeling

Although the role of perceived control has not been explicitly highlighted in the previous literature, the relationship between emotional dissonance and resource depletion is not a new notion. Rather, emotional dissonance has been regarded as a major source of ego-depletion (Baumeister et al., 2007; Deng et al., 2017). The reason behind this relationship is as follows. According to the self-regulation resource theory (Muraven et al., 1998; Vohs and Heatherton, 2000), people use some types of resources to regulate themselves. If an individual is involved in any kinds of regulatory behavior, this uses the resources and leads to depletion (e.g., Schmeichel et al., 2003; Vohs and Schmeichel, 2003). In the context of emotional labor, faking or suppressing emotions requires considerable self-regulatory resource expenditure, which leads to depletion.

Many emotional labor researchers have also examined the relationship between emotional dissonance and a construct very similar to resource depletion under different names such as emotional exhaustion, burnout, fatigue, and energy depletion (e.g., Gross, 1998; Grandey, 2000). Largely, two lines of research stream are noteworthy. In one line of research, emotional dissonance has been found to mediate the effect of emotional labor on emotional exhaustion (e.g., Lewig and Dollard, 2003). In another line of research, emotional exhaustion has been found to mediate the effect of emotional dissonance on job-related outcomes (e.g., Karatepe and Aleshinloye, 2009). These findings together lend a strong support to the relationship between emotional dissonance and emotional exhaustion.

One subtle difference between self-regulation and emotional regulation theory lies in at which level it defines resource depletion (or emotional exhaustion). The latter theory defines emotional exhaustion as the physiological responses while the former theory includes not only physiological but also psychological states. In this research, we take the perspectives of the former.

Then, what would be the consequences of emotional exhaustion? We argue that when people are emotionally exhausted, they have no emotional resources they can use in the interaction with others. As a result, they would be less likely to feel sympathy for others’ feeling. Taken together, we propose that if an individual experiences emotional dissonance, they would feel less sympathy toward others. It is formally stated as,

H1: Those experiencing emotional dissonance (vs. not) would be less likely to feel sympathy for others’ feeling.

### Sympathy and Prosocial Behavior

What, then would be the consequences of this lowered sympathy? A body of literature on prosocial behavior shows the strong linkage between individual’s sympathy (or empathy) and prosocial behavior (Eisenberg et al., 1989; Eisenberg and Fabes, 1990). For example, Eisenberg et al. (1989) measured the degree to which one feels sympathy toward others using various ways including facial expression, physiological index (e.g., heart-rate), as well as self-report. Each measure was shown to be a strong predictor of one’s prosocial behavior, supporting the notion that sympathy and prosocial behavior is strongly linked. Further, Jolliffe and Farrington (2006) administered the 20-item Basic Empathy Scale (BES) to 363 K-10 adolescents (aged about 15) and found that empathy was positively related to prosocial behavior and a lack of empathy associated with aggressive and antisocial behavior. A corollary hypothesis of these finding, along with our H1, is that emotional dissonance would have negative impacts on prosocial behavior. Therefore, we argue that, if people experience (or recall their experiences of) emotional dissonance, they would be less likely to act pro-socially because of lowered sympathy from feeling emotional dissonance. This argument is formally hypothesized as,

H2: Those experiencing emotional dissonance (vs. not) would be less likely to act pro-socially.
H3: The effect of emotional dissonance on prosocial behavior will be mediated by sympathy for others’ feeling.

### Differential Effects of Surface and Deep Acting on Sympathy and Prosocial Behavior

If our proposed effects are driven by emotional dissonance, emotional laborers engaged in deep acting (vs. surface acting) would not exhibit the same pattern of behavior (i.e., lowered sympathy and lowered willingness to act pro-socially). For deep acting, by definition, is a strategy to adapt one’s inner thoughts and feeling (Ashforth and Humphrey, 1993), it is less likely to trigger emotional dissonance because, for those engaged in deep acting, the discrepancy between felt and expressed emotions would be minimal. This would subsequently lead to higher sense of control, and less emotional exhaustion. Hence, we predict that those taking deep acting strategy would be more likely to feel sympathy for others’ feeling (than those taking surface acting strategy) and thus results in higher prosocial behavior intention. This hypothesis is formally stated as,

H4: Different emotion regulation strategies would have differential effects on prosocial behavior intention; those engaged in deep (vs. surface) acting would be more (vs. less) likely to feel sympathy for others’ feeling, and subsequently exhibit higher (vs. lower) willingness to act pro-socially

### Overview of Studies

In a series of multiple studies, we tested our hypotheses. We first report the results of a study that examines the relationship between emotional dissonance and the degree to which one feels sympathy for the needy (Study 1). Then, we report two studies that establish the basic effect. We either manipulated (Study 2A) or measured (Study 2B) individual’s emotional dissonance and found that, the greater dissonance one feels, the less one is willing to help others (Study 2A) or the less money one actually donates to a charity (Study 2B). Finally, we tested if different emotion regulation strategies (i.e., deep acting vs. surface acting) would have differential impacts on prosocial intention (Study 3). Additionally, more refined process measures (e.g., sense of control and emotional exhaustion) are tested with serial mediation tests (Study 3).

## Study 1 Does Feeling Emotional Dissonance Lead to Less Sympathy?

The purpose of Study 1 is to examine the relationship between emotional dissonance and sympathy for others’ feeling. As we stated in our theorizing, previous research predicts that those experiencing emotional dissonance would become less sympathetic for others’ feelings. We test this hypothesis in this study.

### Methods

A total of 201 participants (*M*_age_ = 36.06, 41.8% Women) who claimed themselves as service workers were recruited through Amazon’s mTurk. We divided the participants into two groups and varied the degree to which one feels emotional dissonance. Thus, the experimental design was a simple factor (Emotional dissonance vs. Control) between-subjects design. Participants’ emotional dissonance was manipulated with a writing task. Specifically, we asked half of the participants to vividly recall and write about their own experiences where they had to hide their true feelings because of the guidance imposed by their employer (see Appendix A). This instruction was carefully crafted by combining the definitions of both emotional dissonance and emotional labor proposed by previous literature (Hochschild, 1983). For the other half of the participants, we asked them to write about the place in which they were at the moment (Nikolova et al., 2017; see Appendix A). This group of participants serve as a control group. There were no requirements for or limits on the length or content, but we forced the participants to write at least for one and half minutes to proceed to the next page in the survey by hiding the next button. All participants wrote at their pace, and the average time they spent on this writing task was 2 min and 21 s.

After completing the writing task, all participants were informed that the first study was done. Then, they completed a filler task described as an unrelated second study. Following the filler task, participants answered to a battery of demographic questions into which we ostensibly inserted the six-item ‘Sympathy for the Feelings of Others’ scale (Lee, 2009, see Appendix B for more details). This scale was measured on 7-point Likert scale (1 = Strongly disagree, 7 = Strongly agree) and serves as our dependent variable. Any negatively worded items (e.g., I really don’t get emotional when I see people crying) were reverse-coded in all of the analyses throughout the paper. Thus, the higher score means that people feel more sympathetic for others’ feeling.

### Results and Discussion

Independent *t*-test results showed that there is a significant difference in the sympathy for others’ feeling scores (Cronbach’s α = 0.76) between the two groups (emotional dissonance vs. control). Participants in the emotional dissonance group reported a lower sympathy score (*M* = 3.94, *SD* = 1.30) than those in the control group [*M* = 4.50, *SD* = 1.28, *t*(199) = −3.06, *p* < 0.01]. This result is consistent with and thus support H1 that people experiencing emotional dissonance would be less likely to feel sympathy for others’ feeling. The result of Study 1 provides initial evidence for the notion that emotional dissonance negatively influences sympathy for others’ feeling. In the next study, we examine the consequence of this lowered sympathy – prosocial behavior.

## Study 2A Does Feeling Emotional Dissonance Lead to Lower Willingness to Help Others?

The objectives of Study 2A are twofold. First, we examine if there exists a hypothesized relationship between emotional dissonance and prosocial behavior. We predict that if participants feel emotional dissonance, they would be less likely to act pro-socially. Second, we provide process evidence. We propose that lowered sympathy for others’ feeling would mediate the effect of emotional dissonance on prosocial behavior.

### Methods

Two hundred and five participants were recruited from Amazon’s mTurk (*M*_age_ = 38.31, 52.7% Women). As in Study 1, we randomly assigned participants to two experimental groups (Emotional Dissonance vs. Control). Thus, the design of Study 2A is again a single factor between-subjects design. However, different from Study 1 where only service workers were allowed to participate, we imposed no restrictions on the participants’ qualifications in Study 2A for convenience.

Prior to the study, all the participants were informed that they would be participating in several short studies. The first study was introduced as a writing task. We used the same writing task as in Study 1 to manipulate emotional dissonance. We did not, however, hide the next button nor did measure the time spent on the writing task.

Following the writing task, participants proceeded to the next stage framed as an unrelated second study. In this stage, participants were asked to view two fictitious posters of a non-profit organization. Each poster highlights different causes. One poster depicts a group of children in the classroom, soliciting for help on educating children in underdeveloped countries. The other poster portrays people looking over the area seriously damaged by the earthquake, appealing for help on recovering from a disaster. To make them look real, we put the UNICEF logo in the corner of both posters (see Appendix C).

Participants were presented with one poster at a time. Along with the poster, we measured an individual’s willingness to act pro-socially using the following two questions; ‘how much would you be willing to *donate*?’ and ‘how much would you be willing to *share the poster on social media like Facebook or Instagram*?’ (1 = Very unlikely, 7 = Very likely). Given that prosocial behavior includes a broad category of acts such as helping, sharing, donating, cooperating, and/or volunteering (Brief and Motowidlo, 1986; Penner et al., 2005), there are various ways to measure prosocial behavior. We, however, intentionally chose the above two items for reasons. Among many different forms of prosocial acts that maintain or produce the well-being of others, donation (i.e., giving one’s monetary resources to benefit others) is considered as a way that often entails the highest monetary cost to the self (Twenge et al., 2007). In contrast, sharing or click ‘like’ on social media is regarded as a way to help and support others, with minimal monetary, emotional, and temporal cost to the self (see Wang et al., 2017). Therefore, with these two measures, we hope to investigate the effect on emotional dissonance on the full span of prosocial behaviors.

Finally, we collected the same six-item sympathy for others’ feeling scale (Lee, 2009) within the demographic questions as we did in Study 1. We predicted that, compared to the control condition, participants in the emotional dissonance condition would exhibit lower sympathy for others’ feeling and lower willingness to act pro-socially (i.e., less willing to donate and less willing to share the posters on Social Media).

### Results

Participants in the emotional dissonance condition reported lower sympathy for others’ feeling (*M* = 4.59, *SD* = 1.62) than those in the control condition [*M* = 4.96, *SD* = 1.43, *t*(203) = 1.74, *p* < 0.05, one-tailed], replicating the results of Study 1. Next, we separately analyzed the willingness to donate and the willingness to share scales, but no significant differences emerged. Thus, we averaged the two items to create a composite ‘willingness to help others’ measure (*r* = 0.723). Results showed that, compared to the control condition (*M* = 4.04, *SD* = 1.93), participants in the emotional dissonance condition indicated lower willingness to help others [*M* = 3.53, *SD* = 2.01, *t*(203) = 1.81, *p* < 0.05, one-tailed]. The results do not statistically vary when we analyzed the data separately for each poster or using the repeated-measures ANOVA. In either case, when participants recalled their experiences of emotional dissonance, they expressed lower willingness to help providing children with better education and recovering the areas damaged by a natural disaster (see Table 1). Taken together, the results are consistent with our prediction and thus support H2.

**Table 1:** Sympathy for others’ feeling.

	Emotional dissonance	Control	*t*-value	*P*-value
Children’s education	3.48 (2.07)	3.97 (1.98)	*t*(203) = −1.71	*p* < 0.05
Recovery from Natural Disaster	3.59 (2.10)	4.11 (1.98)	*t*(203) = −1.80	*p* < 0.05

*Higher number means higher sympathy. All p-values were calculated with one-tailed test.*

**FIGURE 1:**
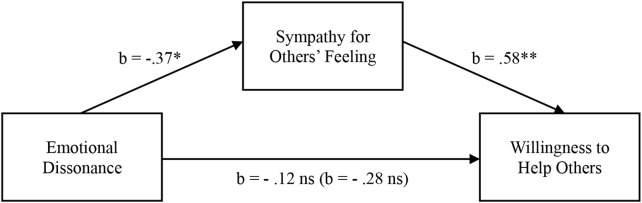
Mediational analysis for Study 2A. ^∗^*p* < 0.05, ^∗∗^*p* < 0.01.

Finally, a mediation analysis (PROCESS model 4 with 5,000 bootstrapped samples, Hayes, 2018) with emotional dissonance as the independent variable, ‘sympathy for others’ feeling’ as the mediator, and the composite measure of ‘the willingness to help others’ as the dependent variable yielded significant mediation via ‘the sympathy for the feeling of others’ (The Bootstrapped Indirect Effect = −.2174, 90% CI = 0.0101, 0.4464) (See Figure 1). This suggests that the negative effect of emotional dissonance on the willingness to help others is mediated by (lowered) sympathy for others’ feeling, which provides support for H3.

### Discussion

The results of Study 2A provide initial evidence for the notion that people experiencing emotional dissonance are less willing to help others. Specifically, we used two different causes (i.e., providing children with better education and recovery from a natural disaster) and two different ways to help (i.e., donation and sharing on social media) to examine the willingness to help others, and the results did not vary by the causes nor by the types of acts, which together increases the generalizability of our findings. Moreover, in Study 2A, we examined the underlying mechanism and found that this emotional dissonance effect is mediated by the lowered sympathy.

It should be, however, noted that the effect sizes reported here are rather weak. We conjecture that this might be due to the fact that we did not impose any restrictions on the qualifications of potential participants. That is, we allowed anyone to participate in our study. Therefore, if a non-service worker was asked to recall their experiences of emotional dissonance, he/she might not be able to vividly recall them, which subsequently may affect our manipulation of emotional dissonance. The data on employment status shows that among 205 participants, only 79% (162/205) were the paid employees (not all of them were service workers), while 14.1% (29/205) and 6.9% (14/205) of the participants were self-employed and not working, respectively. Although we have no data on specific industries in which the participants are working, we posit that the proportion of service workers was lower than 79%, which might contribute to our weak effect sizes. Therefore, hereafter throughout the remaining studies, we collected the data only from the service-workers.

Although the results suggest that emotional laborers (vs. non-emotional laborers) would be less likely to help others, our results in Study 2A are solely based on a self-reported willingness rather than actual behavior. Of course, it is not always required to examine actual behavior in order to generalize the findings on willingness to real behavior because there is a widely accepted notion that attitudes and intention are the precursors of behavior (e.g., Ajzen and Fishbein, 1977). Nevertheless, especially in a prosocial behavior domain, there exists a reason to be more careful in drawing conclusion. Many researchers found that the link between people’s willingness to help and their actual helping behavior is weak (Yoon and Kim, 2018). This implies that non-emotional laborers (i.e., participants in the control condition) who expressed greater willingness to help others actually may not help others more than emotional laborers (i.e., participants in the emotional dissonance condition). Then, the observed between-group difference in the willingness to help others might not be that evident in the real environment, thus practical implications are limited. Therefore, it would be beneficial to examine if the observed emotional dissonance effect can be demonstrated not just in prosocial intentions but also in actual prosocial behavior.

## Study 2B. Do Emotional Laborers Actually Help Others Less?

The purpose of Study 2B is to examine actual prosocial behavior of emotional laborers. For this, we developed a simple experimental paradigm where participants are unexpectedly provided with an opportunity to earn a small amount of money and then determine what percentage of that money they would like to donate to a charity. We employed actual donation behavior because it would be a strong test; the money is real, and the participants have economic incentives not to donate. Therefore, using this experimental paradigm, if we find a difference in the amount of donation by the degree to which one feels emotional dissonance, it would lend more support to our proposition that emotional dissonance negatively affects prosocial behavior.

### Methods

To test if our proposed emotional dissonance effect holds in a situation where the real money is involved, we piggybacked our study on another unrelated survey. We covertly asked three sets of questions at three locations of the unrelated survey; the eligibility questions at the beginning, emotional dissonance questions in the middle, and an extra money offer question at the end. We described these questions more in detail below.

First, the eligibility questions consist of two items about employment status and the specific job functions. If respondents are paid-employees and work in the service-related industry, they are the potential subjects qualified for our study. Second, emotional dissonance is measured with the two-item emotional dissonance questions (Kruml and Geddes, 2000, see Appendix B for more details). Only the qualified respondents saw and answered to the questions on a 5-point scale (1 = never, 5 = always). Finally, the extra money offer question was presented only to them when they reached the last page of the survey after getting the mTurk completion code for the original unrelated survey. The message was titled as ‘Chance for additional $1!’ and asked if respondents want to participate in a 1-min short survey for an extra $1. It was completely their choice to accept the offer. If one wants to participate, they can continue by clicking the ‘yes’ button. Otherwise, they can just opt out by clicking the ‘no’ button.

Once participants have agreed to continue, they were instructed that the purpose of the short survey is to examine how much people would like to donate to a certain non-profit organization. Specifically, they read that they can pledge to donate any percentages of their $1 bonus payment that will be actually matched by us, and their bonus payment will be determined by deducting their pledged amount from the original $1. For instance, if a participant decides to donate 40% of the bonus money (i.e., $.4), a total of $.8 (after we match the amount) will be donated to the charity, and the participant will be receiving $.6 (i.e., $1–$.4). It was emphasized that they can freely choose any percentages between 0 and 100%. Moreover, they read, not to be compelled to look nice, and it would be completely their choice. On the following page, participants viewed a modified version of the UNICEF poster used in Study 2A that solicits donations for children in the underdeveloped country. Participants then indicated what percentages they would like to donate to the charity and what percentages they would like to keep to themselves, respectively, which should sum to 100%.

Participants were recruited through Amazon’s mTurk. Among 1133 mTurkers who accepted the HIT, 301 respondents claimed themselves as the paid-workers in the service industry, thus passed our predetermined criteria. Only these respondents answered to the two-item emotional dissonance questions in the middle of the unrelated survey. Subsequently, it was only to these respondents that the extra money offer question was shown at the end of the unrelated survey. Two hundred forty-four respondents agreed to participate in this extra short survey. Therefore, all of the analyses in Study 2B hereafter are based on these 244 participants (*M*_age_ = 31.36, 31.1% Women).

### Results

We averaged the two emotional dissonance scales (*r* = 0.704) to create a composite measure. Then, it was reverse-coded so that the higher score means the higher felt emotional dissonance. To test our hypothesis on the relationship between emotional dissonance and actual donation behavior, we regressed the pledged percentages to donate (0%∼100%) on emotional dissonance. The result of our regression analysis was consistent with H2. Specifically, those who reported experiencing higher emotional dissonance pledged lower percentages to donate [*Standardized b* = −0.189, *t*(242) = −2.998, *p* < 0.01]. Thus, H2 was supported again in a setting where actual prosocial behavior is engaged.

Although we clearly understand the problems of dichotomizing a continuous independent variable (Fitzsimons, 2008), given that our regression analysis was significant, for illustrative purpose only, we median-split our independent variable (i.e., emotional dissonance) to run ANOVA. A single-factor between-subjects ANOVA yielded the same result; those experiencing high (vs. low) emotional dissonance actually donated less [*M_high_* = 26.45%, *M_low_* = 35.95%, *F*(1,242) = 6.945, *p* < 0.01].

### Discussion

In Study 2B, we replicated the findings of Study 2A that emotional dissonance decreases prosocial behavior. The study design of Study 2B, however, is quite different from Study 2A in several aspects. First, the sample was carefully selected. In contrast to Study 2A where the sample was drawn from the general population, in Study 2B, we drew our sample from the specific group of people that fits our purpose better; paid-employees working in the service-related industry.

Second, different from the previous two studies where we *manipulated* emotional dissonance, we *measured* emotional dissonance in Study 2B. In Studies 1 and 2A, we used the recall-based writing task to manipulate emotional dissonance. Despite our efforts to carefully craft the instructions for emotional dissonance manipulation, the ad-hoc data analysis shows that many participants failed to recall and write about their experiences in the *work context*. Specifically, only 31.2 and 32.7% of participants in the emotional dissonance conditions in Studies 1 and 2A, respectively, described their experienced emotional dissonance in the work place, but the remaining 68.8 and 67.3% described general emotional dissonance experienced in the context of social interaction. Although we believe that it may not debunk our arguments nor change the implications of our findings, we should admit that some constructs (e.g., emotional dissonance) are harder to manipulate than other constructs, and thus manipulation may not be the best way to conduct research on emotional dissonance.

Finally, we used the real money in Study 2B to test our hypothesis. By demonstrating that the results still hold when participants were asked to donate their real money, we showed that our hypothesized effect is very robust. One thing to note here is, the final set of subjects in our Study 2B (*n* = 244) was those who had higher desire for additional money compared with those who had opted out. Therefore, it is reasonable to assume that our sample consists of those the least willing to donate real money. Nevertheless, the result was consistent with our hypothesis; people experiencing higher emotional dissonance pledged to donated 26% less amount of money to the needy.

So far, in three studies, we have demonstrated that those experiencing emotional dissonance are less willing to help the needy, and this effect is mediated by lowered sympathy for others’ feeling. Then, can we conclude that emotional laborers help the needy less? What would happen if an emotional laborer does not experience emotional dissonance much? Does he/she still help others less or more? Our theory suggests that only those experiencing emotional dissonance would be less likely to help others. Therefore, if one is engaged in an emotion regulation strategy that does not lead to emotional dissonance (i.e., deep acting), he/she would not exhibit lowered sympathy nor lowered willingness to help others. We test this idea in the next study.

## Study 3. Differential Effects of Different Emotion Regulation Strategies on Prosocial Behavior

The objectives of Study 3 are twofold. First, as we discussed above, we examine if different emotion regulation strategies would have differential effects on prosocial behavior. We expect that the negative effect of emotion regulation on prosocial behavior would be attenuated in those engaged in deep acting. Second, we test additional mediators between emotion regulation and sympathy for others’ feeling. Following our theorizing, we examine if different emotion regulation strategies affect one’s sense of control and emotional exhaustion differently. By explicitly measuring and testing this theoretically proposed mechanism, we would like to provide with clearer picture of how emotional labor shapes an emotional laborers’ prosocial behavior.

### Participants

We recruited our participants through Amazon’s mTurk. As in Study 2B, we asked two-item eligibility questions at the beginning of the survey, and only those who passed our predetermined qualification (paid-employees working in the service-related industry) participated. We targeted to collect 300 subjects. A total of 2021 mTurkers answered to the eligibility questions until 301 subjects completed our survey (*M*_age_ = 36.11, 46.5% Women).

### Independent Variables

Although emotional dissonance is a concept very closely related to surface acting, and thus they are often interchangeably used in the emotional labor literature, emotional dissonance is distinct from surface acting in the sense that the former arises as resulting experiences from performing the latter (Van Dijk and Brown, 2006). Given that the purpose of Study 3 is to examine the effects of different emotion regulation strategies (not emotional dissonance) on prosocial behavior, what we have to measure is the degree to which an employee uses a certain emotion regulation strategy not the degree to which an employee perceives emotional discrepancy between the felt and expressed emotions. Therefore, the independent variable in Study 3 (i.e., surface and deep acting) should be different from those in previous studies (i.e., emotional dissonance). For this reason, we moved away from the two-item emotional dissonance scales used in Study 2B to surface and deep acting scales in Study 3. We used five items from Grandey (2003) to measure surface acting (Cronbach’s α = 0.91), and four items from Brotheridge and Lee (2003) and Grandey (2003) to measure deep acting (Cronbach’s α = 0.88). They were all measured on 5-point scales (1 = Never, 5 = Always, see Appendix B for details).

### Mediators

Another purpose of Study 3 is to measure additional mediators. According to our theorizing, whether people are engaged in surface or deep acting, their sense of control will be influenced. This perceived control then influences emotional exhaustion, sympathy for others’ feeling, and subsequently pro-social intention. Therefore, the potential mediators we could measure include ‘sense of control,’ ‘emotional exhaustion,’ and ‘sympathy for others’ feeling.’

Participants’ perceived ‘sense of control’ was measured with Yoon and Kim (2018) one item question on a 10-point scale (1 = none at all, little, 10 = a great deal, a lot; see Appendix B for details). ‘Emotional exhaustion’ (Cronbach’s α = 0.95) was measured with nine items adopted from Maslach and Jackson (1981) Burnout scale (1 = Never, 7 = Always). This burnout scales consist of three subscales; emotional exhaustion (nine items), depersonalization (five items), and personal accomplishment (seven items). Although we were not interested in the other two subscales (i.e., depersonalization and personal accomplishment), we just used the whole burnout scale for exploratory purpose^2^. ‘Sympathy for others’ feeling’ (Cronbach’s α = 0.83) was measured with the same six items used in Studies 1 and 2A (Lee, 2009) on 7-point Likert scales (1 = Strongly disagree, 7 = Strongly agree).

### Dependent Variables

We measured our dependent variables using two scales; prosocial behavior (Caprara et al., 2005) and charitable behavior (Charities Aid Foundation, 2012). The former consists of 16 items (Cronbach’s α = 0.95) that encompass a variety of prosocial behaviors from sensing friends’ discomfort (“I immediately sense my friends’ discomfort even when it is not directly communicated to me.”) to lending money (“I easily lend money or other things.”). The latter consists of eight items (Cronbach’s α = 0.89) that also cover various charitable behaviors an individual can do for a non-profit organization. The former was measured on 5-point scales (1 = Never/Almost never true, 5 = Almost always/Almost true) while the latter on 7-point scales (1 = Very unlikely, 7 = Very likely). Each scale was presented to participants with specific instructions (see Appendix B).

### Procedure

All of our independent variables, mediators, and dependent variables were measured in the reverse order to avoid demand artifact. That is, participants answered to the questions about prosocial and charitable behavior first, then about sympathy for others’ feeling, emotional exhaustion, sense of control, and finally about surface and deep acting followed by demographic questions.

### Results

#### Prosocial Behavior

To test our hypothesis, we first ran two simple regressions. When we regressed prosocial behavior on surface acting, surface acting was found to be negatively associated with prosocial behavior [*Standardized b* = −0.118 *t*(299) = −2.057, *p* < 0.05]. This result is conceptually consistent with the findings of Studies 2A and 2B that emotional dissonance leads to lower willingness to help others. In contrast, when we regressed prosocial behavior on deep acting, the opposite pattern emerged. The result shows that deep acting is positively associated with prosocial behavior [*Standardized b* = 0.401 *t*(299) = 7.562, *p* < 0.001]. Taken together, these results show that different emotion regulation strategies (i.e., surface and deep acting) have differential effects on prosocial behavior. Thus, H4 was supported. When we analyzed each item of prosocial behavior separately, the pattern of the results did not vary. Hence, we just report the results with the composite measure hereafter.

#### Charitable Behavior

We ran the same two regressions with charitable behavior as the dependent variable. When we first regressed charitable behavior on surface acting, different from our prediction and findings of Studies 2A and 2B, no significant associations were found [*Standardized b* = 0.046 *t*(299) = 0.799, *p* = 0.425]. We address this unexpected null effect in discussion section below. In contrast, when we regressed charitable behavior on deep acting, deep acting was found to be positively related to charitable behavior [*Standardized b* = 0.390 *t*(299) = 7.321, *p* < 0.001]. Taken together, the results again support the idea that different emotion regulation strategies differentially affect prosocial behavior.

#### Mediational Analyses

To test if the theorized variables serially mediate the effect of emotion regulation strategies on prosocial behavior, we fit the data with model 6 in SPSS PROCESS macro with 5,000 bootstrapped samples (Hayes, 2018). When we ran a mediation analysis with surface acting as the independent variable, ‘sense of control,’ ‘emotional exhaustion,’ and ‘sympathy for others’ feeling’ as the mediators by the order, and the composite measure of ‘prosocial behavior’ as the dependent variable, the indirect effect of ‘surface acting’ on ‘prosocial behavior’ via three mediators was significant (The Bootstrapped Indirect Effect = −0.0029, 95% CI = −0.0077, −0.0003).

The signs of beta coefficients were also all consistent with our theory. Specifically, ‘surface acting’ was negatively associated with ‘sense of control’ [*b* = −0.363, *t*(299) = −2.855, *p* < 0.005]. ‘Sense of control’ was negatively associated with ‘emotional exhaustion’ [*b* = −0.191, *t*(298) = −5.060, *p* < 0.001]. ‘Emotional exhaustion’ was negatively associated with ‘sympathy’ [*b* = −0.152, *t*(297) = −2.643, *p* < 0.01]. ‘Sympathy’ was positively associated with ‘prosocial behavior’ [*b* = 0.273, *t*(296) = 8.886, *p* < 0.001]. These results suggest that when people are engaged in surface acting, they would perceive lowered sense of control, and experience more emotional exhaustion. Emotionally exhausted employees become less sympathetic toward others and subsequently less willing to behave pro-socially. This result again conceptually replicates our main premise that emotional dissonance decreases prosocial behavior via lowered sympathy.

**FIGURE 2:**
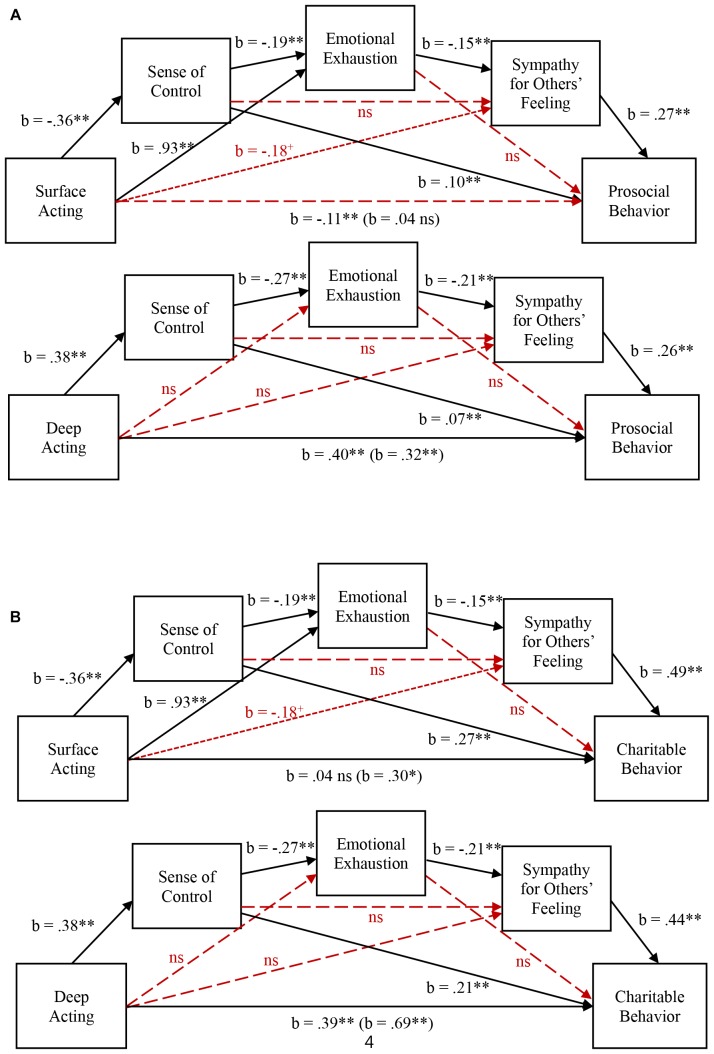
Serial mediational analyses for Study 3. **(A)** The effect of display rules on prosocial behavior. **(B)** The effect of display rules on charitable behavior. ^+^*p* < 0.10, ^∗^*p* < 0.05, ^∗∗^*p* < 0.01.

In addition to this big mediational model, two other mediational paths were also significant. Specifically, the indirect effect of surface acting on prosocial behavior via ‘sense of control’ (The Bootstrapped Indirect Effect = −0.0367, 95% CI = −0.0741, −0.0067), and via ‘emotional exhaustion’ and ‘sympathy for others’ feeling’ (The Bootstrapped Indirect Effect = −0.0387, 95% CI = −0.0732, −0.0102) were significant (see Figure 2A – Any solid lines mean significant paths). The former path indicates that surface acting lowered one’s sense of control, and this lowered perceived control directly influences prosocial behavior. The latter path implies that surface acting (in addition to sense of control) can directly increases emotional exhaustion, and lowered sympathy, then lower willingness to help others.

When we ran a serial mediational analysis with the same dependent variable and mediators but with deep acting as the independent variable, the indirect effect of ‘deep acting’ on ‘prosocial behavior’ via three mediators was again significant (The Bootstrapped Indirect Effect = 0.0056, 95% CI = 0.0009,0.0127). The direct effect of deep acting on prosocial behavior remains significant [*b* = 0.324, *t*(296) = 7.695, *p* < 0.001].

The signs of beta coefficients were also all consistent with our theory. Specifically, ‘deep acting’ was positively associated with ‘sense of control’ [*b* = 0.381, *t*(299) = 2.897, *p* < 0.005]. ‘Sense of control’ was negatively associated with ‘emotional exhaustion’ [*b* = −0.271, *t*(298) = −6.070, *p* < 0.001]. ‘Emotional exhaustion’ was negatively associated with ‘sympathy’ [*b* = −0.214, *t*(297) = −4.380, *p* < 0.001]. ‘Sympathy’ was positively associated with ‘prosocial behavior’ [*b* = 0.256, *t*(296) = 9.155, *p* < 0.001]. These results suggest that when people are engaged in deep acting, they would perceive enhanced sense of control, and be less likely to experience emotional exhaustion. Without emotionally exhausted, employees can be sympathetic toward others and subsequently become more willing to behave pro-socially.

In addition to this big mediational model, one additional mediational path was significant. Specifically, the indirect effect of deep acting on prosocial behavior via ‘sense of control’ (The Bootstrapped Indirect Effect = −0.0279, 95% CI = 0.0035,0.0630) was significant (see Figure 2A). This significant indirect effect indicates that deep acting enhanced one’s sense of control, and this enhanced perceived control directly influences prosocial behavior.

When we ran the same mediational analyses with charitable behavior as the dependent variable, we found very similar results (see Figure 2B), which suggest that surface acting and deep acting influence one’s sense of control in opposite directions and subsequently prosocial and charitable behavior. In summary, the results of Study 3 provide strong support for H4 and theorized mechanism.

### Discussion

The results of Study 3 are consistent with our theorizing as well as the prediction of H4. The effect of different emotion regulation strategies on prosocial behavior is serially mediated by perceived control, emotional exhaustion, and sympathy for others’ feeling. When an individual uses surface acting strategy, the pattern is the same as when one feels emotional dissonance; lowered sympathy and lowered prosocial intention. However, when an individual uses deep acting, the pattern is different from when one feels emotional dissonance; enhanced sympathy and higher prosocial intention. This opposite pattern results from the fact that deep acting does not trigger emotional dissonance, which lends more support for our proposed hypothesis that emotional dissonance is the driver of the effects.

**FIGURE 3:**
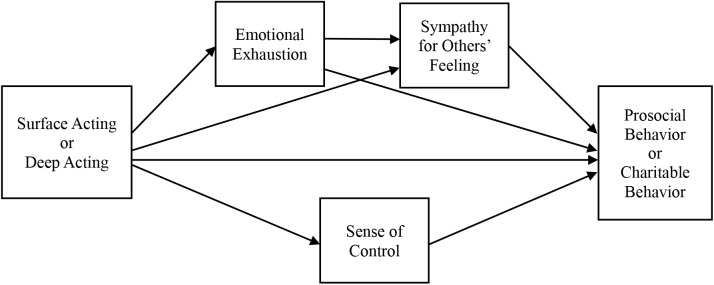
Alternative model configurations for Study 3.

In Study 3, two things are noteworthy. First, when we measured the mediators not explicitly hypothesized but believed to mediate the effect of emotional regulation strategies on prosocial behavior, an interesting pattern emerged; the indirect path from surface or deep acting to prosocial behavior via ‘sense of control’ was significant. It is a very interesting result that could lead to a totally different interpretation and model configuration. When people feel high (vs. low) perceived control due to deep (vs. surface) acting, they would be more (vs. less) likely to help others because they feel powerful (e.g., Inesi et al., 2011). However, another line of research also suggests that people, when feeling lack of control, could donate and help more in order to enhance self-image or to restore illusory control by helping others (e.g., Fiske et al., 1996). We therefore cannot make predictions in one or the other way. Nevertheless, it is possible that sense of control exerts direct influence on prosocial behavior independent of emotional exhaustion. Further, among three mediators tested in Study 3, sense of control seems to be closer to cognitive process while the other two mediators affective. From this reasoning, we explored a different model shown in Figure 3. This model assumes that the effect of emotion regulation strategies on prosocial behavior is mediated by dual paths (i.e., cognitive and affective). When we estimated the model, some of fit indices did not pass the threshold (GFI = 0.84, AGFI = 0.81, CFI = 0.94, RMSEA = 0.05). Hence, we dropped this model.

Second, when we tested the effect of surface acting on charitable behavior, we found no associations between two variables. This result is not consistent with our findings that surface acting negatively influences prosocial behavior. We have gone extra miles to come up with plausible explanations, but our best conjecture is that participants might be influenced by the instructions. That is, when we asked the questions regarding charitable behavior, we asked our participants to think about a non-profit organization, and answer if they would do various charitable behavior. Therefore, people may think about their favorite organization, and probably answer more positively. But there exists no ancillary data to test this conjecture, thus we leave this issue as it is.

## General Discussion

Findings of four studies provide consistent support for the emotional dissonance effect. That is, individuals experiencing emotional dissonance tend to be less sympathetic for others’ feeling, and subsequently less willing and likely to help others. This interesting phenomenon appears to hold true across various operationalizations. For instance, the pattern of results remains the same when emotional dissonance was manipulated (Studies 1 and 2A) or measured (Studies 2B and 3), and when the decision was hypothetical (Studies 2A and 3) or real (Study 2B), and when participants were considering children’s education or recovery from a disaster (Studies 2A and 2B). Moreover, this effect is found to be dependent on one’s emotion regulation strategies (Study 3). When people are engaged in deep (vs. surface) acting, the observed emotional dissonance effect disappeared. Taken together, the proposed emotional dissonance effect seems very robust.

Although we repeatedly replicated our key findings over multiple studies, statistical significance of support for our claim varied across studies. Especially, the results of Study 2A were significant only with a one-tailed test. We therefore conducted meta-analysis to examine (1) if the effect is on average statistically significant and (2) whether the effect sizes across studies are homogeneous. Following the recommendations by Lipsey and Wilson (2001), we tested the first by a *Z* statistic and the second by a *Q* statistic with df = number of studies compared minus one (see Table 2). Table 2 shows the effect of emotional dissonance on a) sympathy for others’ feeling (Studies 1, 2A, and 3) and b) prosocial behavior (Studies 2A, 2B, and 3). Z statistics for both variables indicate that our effects are very robust and statistically significant. Q statistics for both variables are non-significant, which indicates that the null hypothesis of homogeneity cannot be rejected. In summary, the results of meta-analysis suggest that our effects replicated with multiple operationalizations and measures are very robust.

**Table 2:** Meta-analysis of Key Effect of Emotional Dissonance in Studies 1–3.

Effect of emotional dissonance on DVs of interest	Weighted mean effect size (based on Fisher’s Zr)	SE of the mean effect size	*Z*	95% CI Upper bound	95% CI Lower bound	Q	df	*p*-value of Q
Sympathy for others’ feeling (Studies 1, 2A, and 3)	−0.231	0.053	−4.318	−0.126	−0.336	1.324	2	0.516 (n.s.)
Prosocial Behavior (Studies 2A, 2B, and 3)	−0.144	0.043	−3.387	−0.061	−0.228	0.835	2	0.659 (n.s.)

Despite the results of meta-analysis, some may argue that our effects are driven not by the felt emotional dissonance but by different income levels. That is, people are less likely to donate if their income level is low while they are more likely to donate if their income level is high. If the significant results we demonstrated so far were not just spurious correlations (Simon, 1954), it should remain significant when we control for the income. Thus, we tested this possibility for Studies 2A, 2B, and 3. For Study 2A, when we regressed the willingness to help others on both sympathy for others’ feeling and participants’ income, only sympathy for others’ feeling remains significant while income was not. For Study 2B, the unrelated survey did not collect the income data. But we were able to get some data similarly utilized – the last month’s salary. It was sensitive question and thus not forced to answer. As a result, there were many missing values in the Study 2B’s dataset. But when we ran the same regression analysis with only those who answered to the question, the results are basically the same; the salary data was not statistically significant. For Study 3, when we ran the same four regression models in Figure 2. While controlling for income, the patterns of the results were the same and income was also significant positive (0.03 < *b’s* < 0.08, *all p’s* < 0.01). Taken together, we think that the alternative explanation of the income level is not viable.

### Theoretical Implications

To our best knowledge, the present research is the first attempt to examine the effect of emotional labor from the perspectives of consumers. Our research suggests that there exist many unexplored variables beyond the boundary of organizations. For instance, prosocial behavior that we introduced in this research is the variable that has never been examined along with emotional labor. We, however, proposed and demonstrated the significant linkage between emotional labor and prosocial behavior.

Additionally, we proposed multiple theoretical mediators including ‘sense of control.’ By showing that perceived control mediates the effect of emotional dissonance on emotional exhaustion, we unveiled one important construct between emotional dissonance and emotional exhaustion.

Despite not without limitations, we not only measured but also manipulate the degree to which emotional dissonance is perceived.

Research on emotional laborer from the perspectives of consumers has just begun. That means there exist numerous research opportunities in this topic. Especially, it would be fruitful to examine some consumer behavior that can be benefited from perceived emotional dissonance. For instance, if emotional dissonance makes people less emotional, it might be beneficial to consumers in a certain environment where consumers may easily fall prey to strong emotions (e.g., impulsive buying, addiction). The burgeoning area of financial decision making is one of the future areas to which this emotional dissonance research can contribute.

### Practical Implications

Our research suggests that the negative consequences of emotional labor may extend beyond organizational outcomes like Job retention rate or job satisfaction. Although we examined prosocial behavior as our main dependent variable, it is possible that emotional laborers act in ways more detrimental to the society. For instance, if emotional labor makes people to be less sympathetic toward others, it implies that people become more selfish and opportunistic. When more people become selfish, it is obvious that their behavior would incur societal costs. Additionally, it would be more difficult for government or policy makers to encourage people to join the force to solve many problems that should be solved by the society together (e.g., global warming, find dust problems in Asia). Therefore, policy makers may want to establish regulations guiding the display rules imposed by organizations in the service-sector.

Although we are not sure how long the effect of experienced emotional dissonance would last, we found that even briefly reminding of past experiences of emotional labor made people become less sympathetic and less willing to help others. This implies that even after leaving the job, the negative impact of working as a emotional laborer might last for a long time. Therefore, both legislators and policy makers need to think about devising the remedy for this. Guaranteed opportunities for consultation might be one possible solution.

Our findings suggest that deep acting does not lead to lowered prosocial behavior. One quick solution, thus, might be to train employees how to change the way they perceive the situation. Additionally, we found that it is perceive sense of control resulting from emotional dissonance that leads to lowered sympathy and willingness to help others. Thus, if managers could somehow induce this sense of control by various ways, we can expect to reduce the negative consequences of emotional labor.

Companies can improve the situation as well. For instance, increasing number of call centers in South Korea are nowadays allowing customer service workers to end a phone call at their discretion when experiencing verbal abuse (Song, 2017). As many companies in the service sector adopted this policy, it is reported that the well-beings of both companies and employees drastically improved (e.g., number of abusive calls dropped, increased job satisfaction, etc.).

## Conclusion

Our work reported here is, to our best knowledge, the first academic investigation to examine the effect of emotional labor on prosocial intention and actual behavior. We believe that this paper along with our findings shed light on the area that has been neglected; emotional laborers’ behaviors as consumers. As we stated in our introduction, this has significant impact on the society. Our finding suggests that organizations have to carefully design the display rules so that the negative impact of being involved in emotional labor can be minimized. Very often, the focus of organizations can be on just their customers, but their employees need to be considered together for better outcomes for the society.

## Ethics Statement

This study was carried out in accordance with the recommendations of IRB committee at Oklahoma State University with online informed consent from all subjects. The protocol was approved by the IRB committee at Oklahoma State University.

## Author Contributions

Y-nP developed the basic ideas and concepts. All authors designed and ran the experiments together. All analyses were done by Y-nP under the supervision of JJ. Y-nP drafted the manuscript and HH and JJ revised together.

## Conflict of Interest Statement

The authors declare that the research was conducted in the absence of any commercial or financial relationships that could be construed as a potential conflict of interest.

## Footnotes

1 It should be noted that this lowered sense of control would be observed only in the employees engaged in surface acting. If an employee is engaged in deep acting, he/she would not recognize any discrepancy between the felt and expressed emotions (i.e., no emotional dissonance). Therefore, they would perceive that they have control over the way they express their true feeling, which results in enhanced sense of control. In fact, previous research has suggested that deep acting promotes a sense of control (e.g., Brotheridge and Lee, 2002; Scott and Barnes, 2011).

2 Later we analyzed the data including the other two subscales and found that those two subscales are also significant mediators in some model specifications, however, we did not include these measures and would not discuss any further.
