# Supplementary material for: Do Emotional Laborers Help the Needy More or Less? The Mediating Role of Sympathy in the Effect of Emotional Dissonance on Prosocial Behavior
Source: Front Psychol. 2019 Feb 7;10:118. doi: 10.3389/fpsyg.2019.00118 (PMC6374704; doi:10.3389/fpsyg.2019.00118)

Appendix. A - Emotional Dissonance Manipulation (Study 1 and 2A)

Instruction for the Emotional Dissonance condition

Please vividly recall (or imagine) and write about a situation where you had to put active efforts to change or control your emotions to meet social guidelines so that you felt a big difference between your felt and feigned emotions. For instance, while you are working (or meeting) with people, you may have pretended to have certain emotions that you didn't really have or you hid your true feelings about a person or situation. Please feel free to write about that moment and how you felt.

Instruction for the Control condition

Please write a short paragraph describing the room in which you are currently.

Please be thoughtful as you write and describe the room or place where you are now with as many details as possible. Please feel free to write anything about your place.

Appendix B. Measures and Scales Used in Study 1, 2A, 2B, and 3

**Emotional Dissonance** (Kruml and Geddes 2000)

1. I show the same feeling to customers that I feel inside. (R)
2. The emotions I show the customer match what I truly feel. (R)

(1 = Never, 5 = Always)

**Sympathy for the Feelings of Others** (Lee 2009)

1. I really don’t get emotional when I see people crying. (R)
2. It’s common for me to become teary eyed or close to crying when I see others crying.
3. I don’t tend to have feelings of sorrow or concern when I see others crying. (R)
4. I don’t usually get emotional when others around me feel embarrassed or ashamed. (R)
5. I’m inclined to feel really troubled when someone I know is crying.
6. It doesn’t bother me very much when sensitive people get their feelings hurt. (R)

**Surface acting** (Grandey 2003)

1. Just pretend to have the emotions I need to display for my job.
2. Put on an act in order to deal with customers in an appropriate way.
3. Resist expressing my true feelings.
4. Pretend to have emotions that I don't really have.
5. Hide my true feelings about a situation.

(1 = Never, 5 = Always)

**Deep acting** (1-Grandey, 2003 & 2,3,4-Brotheridge and Lee,2003)

1. Work hard to feel the emotions that I need to show to others.
2. Try to actually experience the emotions that I must show.
3. I make an effort to actually feel the emotions that I need to display to others.
4. I really try to feel the emotions I have to show as part of my job.

 (1 = Never, 5 = Always)

**Emotional Exhaustion** **(**Maslach and Jackson,1981)

1. I feel emotionally drained from my work.
2. I feel used up at the end of the workday.
3. I feel fatigued when I get up in the morning and have to face another day on the job.
4. Working with people all day is really a strain for me.
5. I feel burned out from my work.
6. I feel frustrated by my job.
7. I feel I'm working too hard on my job.
8. Working with people directly puts too much stress on me.
9. I feel like I'm at the end of my rope.

**Depersonalization** **(**Maslach and Jackson,1981)

1. I feel I treat some people as if they were impersonal objects.
2. I've become more callous toward people since I took my current job.
3. I worry that my job (has been or) is hardening me emotionally.
4. I don't really care what happens to some people.
5. I feel people blame me for some of their problems.

**Personal Accomplishment** **(**Maslach and Jackson,1981)

1. I can easily understand how my recipients feel about things.
2. I deal very effectively with the problems of my recipients.
3. I feel I'm positively influencing other people's lives through my work.
4. I can easily create a relaxed atmosphere with my recipients.
5. I feel exhilarated after working closely with my recipients.
6. I have accomplished many worthwhile things in this job.
7. In my work, I deal with emotional problems very calmly.

(1 = Never, 7 = Always)

**Sense of Control (**Yoon and Kim, 2018)

1. How much control do you feel you have over the way your life turns out?

(1 =  none at all, little; 10 = a great deal, a lot).

**Prosocial Behavior (**Caprara, Steca, Zelli and Capanna,2005)

The items reflect behaviors and feelings that can be traced back to one of four types of actions, namely, sharing, helping, taking care of, and feeling emphatic with others and their needs or requests.

1. I am pleased to help my friends/colleagues in their activities.
2. I share the things that I have with my friends.
3. I try to help others.
4. I am available for volunteer activities to help those who are in need.
5. I am emphatic with those who are in need.
6. I help immediately those who are in need.
7. I do what I can to help others avoid getting into trouble.
8. I intensely feel what others feel.
9. I am willing to make my knowledge and abilities available to others.
10. I try to console those who are sad.
11. I easily lend money or other things.
12. I easily put myself in the shoes of those who are in discomfort.
13. I try to be close to and take care of those who are in need.
14. I easily share with friends any good opportunity that comes to me.
15. I spend time with those friends who feel lonely.
16. I immediately sense my friends’ discomfort even when it is not directly communicated to me.

(1 = Never/Almost never true, 5 = Almost always/Always true)

**Charitable behavior (**Charities Aid Foundation 2012)

Please think about a non-profit organization that comes to your mind. Then, please indicate on the following scale how likely you would do the following behaviors.

1. Donate money
2. Volunteer time to assist with administrative tasks.
3. Volunteer time where your skills best fit with the organization's goals.
4. Donate supplies that are relevant for the organization to achieve its goals.
5. Donate blood
6. Sign a petition to increase funding for this organization.
7. Spread the word about this organization to others (e.g., share on Facebook, Twitter, etc.; tell family, friends, co-workers, etc.).
8. Ask other people to sign a petition to increase funding for this organization.

(1 = Very unlikely, 7 = Very likely)

Appendix C. Experimental Stimuli (Study 2A and 2B)

**Every child deserves a fair chance in life.**

**Your donation makes a difference for children around the world.**
 
 
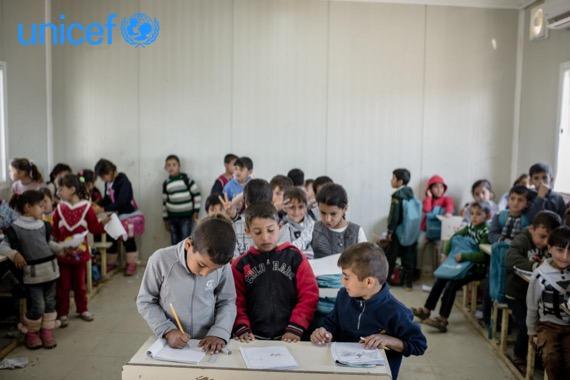


**Support  earthquake disaster recovery in Chiapas, Mexico!**
**Your donation makes a different for them around the world.**

 
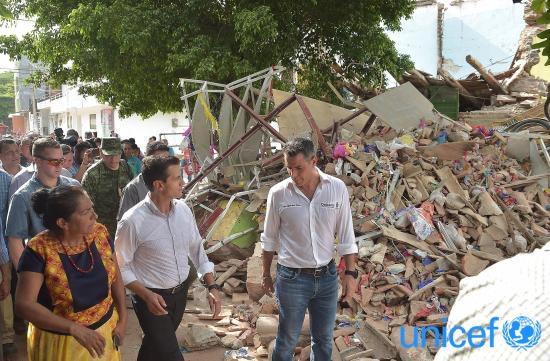

Supplement: Supplementary file 1 [file Data_Sheet_1.docx]
